# Experiences of participation in activities among girls with juvenile idiopathic arthritis: A qualitative study

**DOI:** 10.1177/13674935221083167

**Published:** 2022-04-10

**Authors:** Johanna Kembe, Malin Regardt

**Affiliations:** 1 Women´s Health and Allied Health Professionals Theme, 59562 Medical Unit Occupational Therapy and Physiotherapy, Karolinska University Hospital, Stockholm, Sweden; 2 Department of Women’s and Children’s Health, 27106 Karolinska Institutet, Stockholm, Sweden; 3 Department of Neurobiology, care sciences and society Division of Occupational Therapy, 27106 Karolinska Institutet, Stockholm, Sweden

**Keywords:** arthritis, juvenile [MeSH], occupational therapy [MeSH], leisure activities [MeSH], activities of daily living [Cinahl], social participation [Cinahl]

## Abstract

This study aimed to describe how girls with juvenile idiopathic arthritis (JIA) experience participation in activities in relation to their disease. Semi-structured interviews of eight girls with JIA in ages 12–15 were recorded and transcribed as a whole. Data have been analysed with qualitative content analysis. Participation was influenced by disease symptoms, which could result in absence and exclusion from participation in activities. The experience of participation was affected by the participant’s choice of using strategies and making adjustments during activities. Social environment could both increase and decrease participation through treatment and type of knowledge about the disease. Also the physical environment could affect participation due to the climate or road and ground conditions. We could conclude that the experience of participation among girls with JIA was affected by disease symptoms, perceptions of selves, disease and the use of strategies, and also the social and physical environment. This indicates the need to address participation in clinical practice and the importance to include different health professionals, teachers and other personnel involved in the child’s schooling.

## Introduction

Participation is defined as an individual’s engagement in work, play and activities of daily living which are part of the individual’s context and is demanded and/or necessary for well-being (Kielhofner, 2008a). Participation is characterised by meaningfulness (Yerxa, 1980) and the subjective experience of being part of and belonging to a context (Christiansen et al., 1995; Law et al., 2006; Nelson, 1988).

Juvenile Idiopathic Arthritis (JIA) is a paediatric rheumatic disease characterised by chronic inflammation in the musculoskeletal system that can cause pain, swelling and joint stiffness (Petty et al., 2016) and also fatigue and problems with sleeping (Kutcha and Davidson, 2016). JIA is more common in girls than boys (2:1) (Petty et al., 2016). The prevalence of JIA in Sweden is 14 in 100,000 children (Berntson et al., 2003).

Pain is a significant problem in JIA (Rapoff and Lindsley, 2016) and one of the greatest challenges children face is that pain is subjective and may not be apparent to peers or others (Tong et al., 2012). Juvenile Idiopathic Arthritis may negatively affect activity performance, quality of life and participation in activities (Duffy and Feldman, 2016).

Participation in children with JIA is affected during social, leisure and physical activities and at school (Lelieveld et al., 2008; Maggio et al., 2010; Packham and Hall, 2002; Schanberg et al., 2003). If children with JIA are inhibited from participation, it entails a higher risk for not developing social and cognitive skills, not acquiring the characteristics necessary for adulthood and an increased risk of developing mental illness (Mullick et al., 2005; Reiter-Purtill et al., 2003).

Several studies based on quantitative research show that children with JIA are affected during participation in activities. However, there is a lack of studies investigating the children’s own perception of participation using a qualitative approach. The present study will contribute to the knowledge about children with JIA own experiences of participation in daily activities.

## Aim

To increase the knowledge how children with JIA experience participation in activities in relation to their disease.

## Materials and methods

### Design

This is a qualitative study using content analysis to better understand how children with JIA experience participation in daily activities.

### Inclusion criteria

Children with JIA between 12–15 years of age who had over the last 6 months reported moderate (≤75.70) to severe (≤67.18) impact on quality of life according to the self-assessment questionnaire DISABKIDS. DISABKIDS measures quality of life affected by a chronic disease (The DISABKIDS Group Europe, 2006) and it was assumed that quality of life affected by the disease could also impact participation in daily activities.

#### Exclusion criteria

Children were excluded if they were patients to the authors, had other diagnoses that could affect participation or were not speaking Swedish.

### Recruitment

Children were identified according to an appropriate selection between August 2016 and January 2017 through the Swedish Paediatric Rheumatology Registry.

### Data collection

A letter with information about the study and a consent form were sent to eligible participants and their guardians. The guardians were contacted by telephone 10–14 days after the letter was sent and those who gave an oral consent were booked for interviews. All the guardians and participants of 15 years of age signed the consent prior to the interview. Qualitative semi-structured interviews were conducted by the first author and all interviews were recorded. The semi-structured interview included questions regarding how meaningful activities were performed at home, at school and during leisure. How satisfied they were with the performance of daily activities. Cause and strategies used when not able to perform activities also in comparison to friends and siblings. The participant chose the location of the interview and the attendance of a guardian. Interviews were conducted between October 2016 and January 2017.

The study was approved by the Regional Ethics Review Board in Stockholm, Sweden, registration number 2016/1398-31/1 and was conducted according to the Helsinki Declaration (World Medical Association Inc., 2015).

### Data analysis

The interviews were transcribed in their entirety to 181 pages of text by the first author and a content analysis was applied (Graneheim and Lundman, 2004). At first the interviews were read to get an understanding of the participants description of their experience of participation in activities. Thereafter the text was divided and condensed into smaller parts, sentence units, without losing its meaning. Then the condensed sentence units were labelled with codes and grouped into categories with corresponding sub-categories. Finally, the theme that answered to the aim of the study was made. The main analysis was made by the first author and the results validated through a consensus discussion with the last author to assure trustworthiness. To ensure credibility of the results, quotes were selected from the interviews (Graneheim et al., 2017). Pseudonyms have been used for the participants in the findings.

## Findings

A total of 34 children with JIA were identified through the registry. Fourteen children (nine girls, five boys) declined participation without known reason, seven children (six girls, one boy) could not be reached within the study period. Interviews were performed in an ongoing process until no new experiences were added from the participants. For that reason, five children were not invited to participate. A total of eight girls participated in this study. The median (IQR) age was 15 (1) years with a and disease duration of median (IQR) 7,5 (10) years. Median (IQR) age at diagnosis was 7.5 (9) years. One of the participants had moderate impact on quality of life measured by DISABKIDS and the remaining severe impact. All participants were treated with anti-rheumatic drugs.

The interviews lasted in median 41 min. All participants chose the Occupational Therapy unit at the university hospital as location and a guardian were present in three interviews.

**Figure 1.**
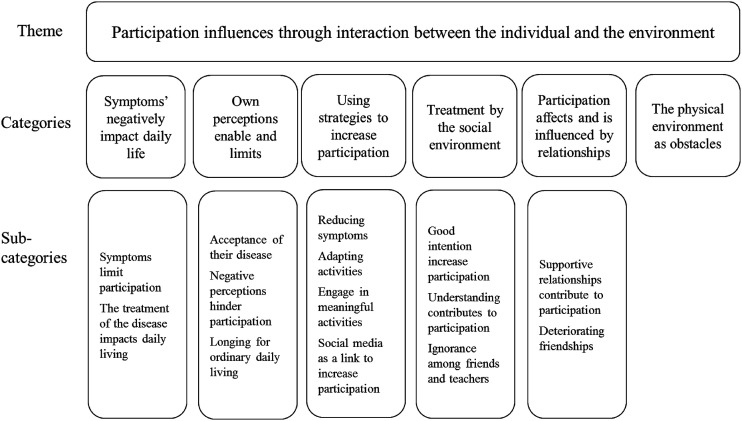
Description of the theme, categories and corresponding sub-categories.

Based on the collected data the theme **Participation influences through interaction between the individual and the environment** with corresponding categories *Symptoms’ negatively impact daily life*, *Own perceptions enable and limits*, *Using strategies to increase participation, Treatment by the social environment, Participation affects and is influenced by relationships* and *The physical environment as obstacles,* was formed with associated sub-categories (Figure 1).

### Symptoms’ negatively impact daily life

The participants described that the symptoms both directly and indirectly impacted participation during physical and social activities and at school.

#### Symptoms limit participation

Experience of symptoms such as pain, fatigue, stiffness and reduced energy could lead to absence from school and difficulties participating in social and structured activities with friends after school. The symptoms the participants experienced could cause difficulties concentrating which led to difficulties doing assignments at school and homework. One symptom could lead to other symptoms and it became a vicious circle. This subcategory can be described with this quote:

“And then after school I am often very tired and it becomes difficult to do homework and I feel that I have no time for anything else than doing homework /…/ Because I have no time to be with friends and so /…/ I cannot take it.” (Sara)

#### The treatment of the disease impacts daily living

Participation was indirectly affected by increased symptoms caused by insufficient pharmaceutical treatment or when the disease flared. Also, the need to attend health care appointments often resulted in school absenteeism and the need to catch up on schoolwork at home. One participant expressed:

“Because of /…/ the doctor appointments /…/ then it is a bit difficult to do it (homework). And then I tell my teachers “yes sorry”. /…/ “I am sorry I couldn’t, I had an appointment to the doctor”. (Hanna)

### Own perceptions enable and limits

Participation was affected by the participants own acceptance and attitudes towards the disease, as well as their negative perception and anxiety about how the disease influenced daily living. The participants described that their perceptions could both increase and decrease the experience of participation, and the participants expressed how they were wishing for working routines in their daily living.

#### Acceptance of their disease

The experience of participation increased when the participants had accepted their disease and their overall situation. A sense of satisfaction was expressed by the participants when they could engage in meaningful activities and a sense of pride when there was a functioning everyday life. Not feeling sorry for oneself increased participation and performance during activities. One quote describing the subcategory is:

“So I like thinking there is nothing that I miss and I have like accepted that this is how it is going to be.” (Sara)

#### Negative perceptions hinder participation

The participants’ negative thoughts and perceptions, attitudes, fears and concerns about their own abilities and diseases decreased the experience of participation in daily living. Even though the participation decreased, the participants expressed that they would refuse to use technical aids because they thought it was embarrassing and were worried that their friends would exclude or treat them badly. They expressed being worried to be associated with a disease and being treated differently by teachers that could make their classmates jealous. They were worried about being substituted during team sports because of their disease and not to be able to take part like classmates. The participants were unwilling to be different and wanted to be like everyone else. The following quote reflects this subcategory:

“No but like everyone has kind of started to chat with boys, started dating boys. Then it’s not fun to have a wheelchair, it’s not possible. We are going out doing things, I would never.” (Linda)

#### Longing for ordinary daily living

The participants expressed wanting to be well and engage in normal daily living with ordinary routines at school and during leisure in order to experience continuous participation during social activities with friends. This subcategory can be described with this quote:

“Like, be more like social and just /…/ I think it’s really fun, or like, participate during activities like sports.” (Carin)

### Using strategies to increase participation

Different strategies to enable participation in activities and to increase participation was used by the participants.

#### Reducing symptoms

By using strategies to reduce symptoms the participation increased. These strategies included the use of pain-relieving drugs, sleeping to reduce pain and gain energy and mobility exercises, as well as wearing warm clothes and using heating aid to prevent stiffness and pain. One participant expressed:

“So it hurts like I have to get up and kind of move because it gets like stiff.” (Julia)

#### Adapting activities

The participants described that their activities were adapted to increase and maintain participation*.* Participation during social activities, at home and at school increased when they used technical aids. During walks or when visiting a shopping centre a wheelchair or crutches were used to improve mobility and also participation. At school the participants expressed that the use of computer, tablet or pen grip decreased pain when writing. The activities had to be adapted and planned based on their daily fitness and level of pain, fatigue and energy. This resulted in adaption at school, during sports lessons, their choice of leisure and physical activities, and the level of intensity during activities. One quote describing the subcategory is:

“After the autumn during 8^th^ grade I got an iPad that I used for a while… it hurt writing.” (Sanna)

#### Engage in meaningful activities

Activities that were motivating, important and enjoyable enabled participation and could be performed regardless if the symptoms increased. The participants prioritised participation in meaningful activities at school, physical activities and social activities. One participant described their prioritisation with this quote:

“Just if I think something is very fun and I have decided to do it /…/ then I am ignoring if there is pain. Then I will do it because I think it’s fun.” (Carin)

#### Social media as a link to increase participation

The possibility of staying in contact with friends via social media when they were unable to attend school or join in activities increased the participation and feeling of being part of a social context. This subcategory can be described with this quote:

“Yes, but if you don’t go to school one day you can just get in touch there. So, no but it’s very important.” (Carin)

### Treatment by the social environment

The participants expressed that treatment by the social environment affected the experience of participation both negatively and positively.

#### Good intension increase participation

Caring and thoughtful treatment from family and friends experienced the participants contributed to a sense of social belonging. The sense of social belonging increased participation in activities even though the participants might not be active during the activity. One participant expressed:

“So they (classmates) are still very nice, they are always like this asking /…/ can you walk this fast or should we walk slowly? /…/ they still think in a way so.” (Carin)

#### Understanding contributes to participation

The participants expressed that their participation and activity performance increased when their family, friends, teachers and friends with JIA or other disabilities understood the consequences from the disease. To have a friend with JIA felt authentic and was an uncomplicated friendship where the friends understood each other’s situation. This subcategory is reflected by the following quote:

“The others they understand (friends with JIA), they know if you say that it feels like this. Then they know exactly how it feels. So it’s very nice.” (Sara)

#### Ignorance among friends and teachers

Experience of participation decreased among the participants when they were treated with ignorance and a lack of understanding regarding their recourses and limitations during activities because of the disease. Not being understood regarding the need to spend more time performing activities or adjusting activities by using a technical aid or transportation expressed the participants as frustrating and tedious. The participants were worried being labelled lazy and not wanting to participate in activities. When friends misunderstood how the disease affected the participants it could result in them not being included in activities. However, ignorance of the disease among friends could also result in increased participation because the friends did not understand how the disease affected the participants, and the participants were treated like anyone else. One quote describing the subcategory is:

“Ehm but other friends eh…I don’t really think that they understand because… like it’s so unusual. It can be an hour when I cannot walk and the other hour I can sort of run. It’s, it’s difficult to understand. I don’t even understand myself.” (Amina)

### Participation affects and is influenced by relationships

The participants expressed that to socialise with people who were accepting and supportive increased their ability to participate, and the participants varying abilities to take part in activities affected participation.

#### Supportive relationships contribute to participation

Relationships, social belonging and acceptance are of great importance for participation. When interacting with close friends, friends with JIA or friends with other disabilities participation increased. This resulted in experience of social belonging, cohesion and acceptance of the disease, activity capacity and performance. To be supported by friends with JIA that had experience of the disease, gave support and motivated them to take part in activities. One participant described her supportive friends:

Because my friends are always like this /…/ they don’t care if I have a wheelchair or not because they want like, they take me for what I am. /…/ that is nice. (Carin)

#### Deteriorating friendships

Friendships could be negatively affected due to the participants varying abilities to take part in activities. Not be able to fully engage at school and structured physical activities, resulted in a loss of social activities and deteriorated friendships. This subcategory can be described with the following quote:

“I would not like call the classmates and be with them on my spare time /…/ I don’t have such a good relationship with them since I don’t meet them every day.” (Carin)

### The physical environment as obstacles

According to the participants, the physical environment could be a barrier to participation both at school and during social activities. To transfer could be difficult regardless if technical aids were used or not. For instance, the participants expressed that the road and ground conditions mattered for the ability to move and participate. The participants were affected by the weather, and indoor and outdoor temperature because this could cause stiff and painful joints. One participant explained how the environment affected participation:

“… if there were several who did not want to go skiing. Eh and of course I want to go there and so, but maybe I cannot go as much because it’s partly cold.” (Sanna)

## Discussion

Finding from the present study have enhanced the knowledge, from the children perspective, how participation is influenced by JIA in girls. Participation in activities was influenced by disease symptoms, which could result in absence and exclusion from participation. The individual’s attitude to using strategies and making adjustments during activities led to increased participation and those who chose not to experienced decreased participation. The social environment could increase participation through good treatment and understanding, but the social environment could also decrease participation through misunderstandings and misinterpretation of the disease.

According to present study, disease symptoms affected daily living and caused a decreased participation at school, during social and physical activities. This corresponds with several studies (Armbrust et al., 2016; Cavallo et al., 2015a, 2015b; Maggio et al., 2010; Oliveira et al., 2007; Packham and Hall, 2002; Race et al., 2016; Schanberg et al., 2003). Pain is a significant problem in JIA and pain can cause decreased participation at school (Nordal et al., 2019; Rapoff and Lindsley, 2016; Schanberg et al., 2003). The present study illustrates other symptoms such as tiredness, reduced energy and stiffness affecting participation at school, and has provided new knowledge that symptoms can affect the ability to concentrate at school.

Participation increased when the participants accepted their disease, activity performance and overall situation. However, participation decreased by negative attitudes and feelings and a fear of the disease. The participants had a desire to be normal, which can be found in children with disabilities whom wanted to perform activities on the same terms as their friends (Pereira et al., 2010). The participants were longing for daily routines, but this could be difficult since children with JIA can experience fluctuating symptoms and their ability to perform activities can vary greatly (Tong et al., 2012).

Living with a chronic disease such as JIA can cause psychological and emotional strain and affect the mood, which can impact participation at school and social activities (Mullick et al., 2005; Shaw et al., 2006). The participants in the present study chose not to use technical aids because of other people’s behaviour, even though it would decrease their participation. This can be found in children with disabilities (Kielhofner, 2008b).

The participants experienced difficulties taking part in activities generally and regularly which has been demonstrated in a previous study (Cavallo et al., 2015a). The participants needed to adapt activities at school, during sports lessons, leisure and physical activities to maintain and increase participation. Without adaption they could experience a decreased of participation. The findings correspond with other studies showing that participation for children with JIA was affected when activities were not adapted (Cavallo et al., 2015b) and when adapting activities at school participation increased (Livermore et al., 2016; Race et al., 2016). The participants had different strategies for adapting activities. However, the need to adapt can induce negative feelings and outcomes when performing an activity and participating because they cannot behave as previously (Kielhofner, 2008b). This was not found in the present study. Although it can be imagined that the participant’s unwillingness to adapt activities, use technical aids and not stand out could be related to adaptation having a negative effect on an individual and their self-esteem. This can be found in a previous study in which children with JIA did not want to appear abnormal and therefore chose not to adapt activities at school (Livermore et al., 2016).

Social media was used as a strategy for participation and this strategy is found in studies on children with disabilities (Whiteford, 2010). The children used websites, forums, blogs and interactive games to increase their participation and reduce social isolation (Whiteford, 2010). According to present study participation increased when using social media together with friends when unable to attend activities. It can be assumed that social media was used as a link for interaction with friends as well as being part of a social belonging and context.

Thoughtfulness and understanding from the social environment were of importance to participation. Participation increased when shown consideration and understanding towards one’s capacity to perform activities and the need to adapt activities. According to the International Classification of Functioning, Disability and Health (ICF), participation is affected by the social environment with family, friends and teachers (WHO, 2001). In the present study, decreased participation was experienced when there was ignorance and misunderstanding among family and teachers, which might result in exclusion from activities. The feeling of not being understood was found in a study on children with disabilities (Pereira et al., 2010) and on children with JIA (Livermore et al., 2016), but without a link to the experience of participation.

Children with disabilities experience participation when interacting within their social environment (Pereira et al., 2010). The children experienced participation if the social environment was supportive, if activities were performed on the same terms as their friends, if they engaged in parts of the activity by contributing as a whole, and also watched the activity but contributed with their involvement. A loss of participation occurred when the children could not perform the activity or when they felt socially excluded (Pereira et al., 2010). This corresponds with the present study where the participants experienced participation when the social environment was supportive and understanding, while unwanted treatment affected participation and made the participants feel abnormal.

The physical environment affected the availability of activities with or without technical aids, as did the climate and temperature. Corresponding results in adults with Rheumatoid Arthritis indicate that climate affect participation in physical activities (Qvarfordt et al., 2019). According to the ICF the environment affects activities and participation (WHO, 2001). Indicating the importance to include the environment when addressing participation in JIA.

## Limitations

The present study has some limitations. Qualitative studies involve a small number of participants but are rich in depth. It is not possible to generalise the results to a broader population but there are opportunities to gain new knowledge in areas that are lacking, as is the case in the present study.

The participants were identified through the Swedish Pediatric Rheumatology Registry and the selection of participants was based on DISABKIDS (The DISABKIDS Group Europe, 2006). It is regarded as a strength that selection from the registry was made for a broader variation of participants. One limitation of this study is that only girls participated, which does not represent the gender distribution of the disease. This affects the transferability (Graneheim and Lundman, 2004). Results from a study on children with JIA indicated that older children engaged in more social activities and girls found them more enjoyable than boys (Cavallo et al., 2015a); however, this could not be addressed in the present study.

Another strength of the study was that the participants were of different ages, had varying disease duration which increases the study´s credibility (Graneheim and Lundman, 2004). The choice of DISABKIDS as a basis for the recruitment of participants was based on the authors’ preunderstanding that children who experienced a reduced quality of life may also experience participation restriction in activities. It is important to not let professional experiences and interests affect the outcome of the study. Therefore, reflection on preconceptions has been maintained during the research process.

The first author, who conducted the interviews and parts of the analysis process, has several years of experience and knowledge of working with children with JIA. This could be considered a weakness and may have affected the outcome. However, being able to ask appropriate interview questions and follow-up questions requires knowledge and an interest in the topic. The authors of the study have discussed the data and outcome continuously during the analysis process to ensure consensus and to achieve credibility (Graneheim and Lundman, 2004; Hsieh and Shannon, 2005).

## Implications for practice

The finding indicates the need to address participation in clinical practice in children with JIA where an occupational therapist with expertise in activity and occupational science could help to improve participation in daily life with tools to manage the disease in individually based interventions. It is of importance that children with JIA have contact with a multidisciplinary team since the disease affects the children’s daily living and development. Also, teachers and other personnel involved in the child’s schooling is important to include.

## Conclusion

In conclusion, the participants in the present study experienced participation in activities were affected by the disease symptoms, which resulted in limitations and exclusion from participation. The physical environment could affect participation negatively and the social environment could both increase and decrease participation through treatment, ignorance and level of understanding. The participants’ own approach to using strategies and adapting their activities resulted in increased participation, whereas those who chose not experienced decreased participation. The findings indicate the need to address participation in clinical practice and the importance to include different health professionals and also teachers and other personnel involved in the child’s schooling.
